# MCMV-mediated Inhibition of the Pro-apoptotic Bak Protein Is Required for Optimal *In Vivo* Replication

**DOI:** 10.1371/journal.ppat.1003192

**Published:** 2013-02-28

**Authors:** Peter Fleming, Marc Kvansakul, Valentina Voigt, Benjamin T. Kile, Ruth M. Kluck, David C. S. Huang, Mariapia A. Degli-Esposti, Christopher E. Andoniou

**Affiliations:** 1 Immunology and Virology Program, Centre for Ophthalmology and Visual Science, The University of Western Australia, Nedlands, Western Australia, Australia; 2 Centre for Experimental Immunology, Lions Eye Institute, Nedlands, Western Australia, Australia; 3 Department of Biochemistry, La Trobe University, Melbourne, Victoria, Australia; 4 The Walter and Eliza Hall Institute of Medical Research, Parkville, Victoria, Australia; 5 Department of Medical Biology, University of Melbourne, Melbourne, Victoria, Australia; University of Alberta, Canada

## Abstract

Successful replication and transmission of large DNA viruses such as the cytomegaloviruses (CMV) family of viruses depends on the ability to interfere with multiple aspects of the host immune response. Apoptosis functions as a host innate defence mechanism against viral infection, and the capacity to interfere with this process is essential for the replication of many viruses. The Bcl-2 family of proteins are the principle regulators of apoptosis, with two pro-apoptotic members, Bax and Bak, essential for apoptosis to proceed. The m38.5 protein encoded by murine CMV (MCMV) has been identified as Bax-specific inhibitor of apoptosis. Recently, m41.1, a protein product encoded by the m41 open reading frame (ORF) of MCMV, has been shown to inhibit Bak activity *in vitro*. Here we show that m41.1 is critical for optimal MCMV replication *in vivo*. Growth of a m41.1 mutant was attenuated in multiple organs, a defect that was not apparent in *Bak^−/−^* mice. Thus, m41.1 promotes MCMV replication by inhibiting Bak-dependent apoptosis during *in vivo* infection. The results show that Bax and Bak mediate non-redundant functions during MCMV infection and that the virus produces distinct inhibitors for each protein to counter the activity of these proteins.

## Author Summary

The cytomegaloviruses (CMV) are a family of viruses that establish a latent infection that lasts for the life of the host, with the virus able to reactivate when the host is immunosuppressed. We have used murine CMV (MCMV) as a model to understand how CMV interferes with the anti-viral immune response. Apoptosis, or programmed cell death, is one of the defence mechanisms used by multicellular organisms to impair viral infection. In order for viral replication to proceed, many viruses have evolved mechanisms to prevent the apoptosis of infected host cells. Under most circumstances the activation of Bax, or the closely related protein Bak, is required for apoptosis to proceed. The m41.1 protein was recently identified as a candidate Bak inhibitor during *in vitro* infection. We have generated a mutant virus which is unable to produce the m41.1 protein and found that growth of this virus was attenuated in wild-type mice. Importantly, growth of the mutant virus was equivalent to that of the wild-type virus in mice lacking the Bak protein. These studies establish that m41.1 is an inhibitor of Bak and that the capacity to prevent apoptosis triggered by Bak is required for efficient replication of MCMV *in vivo*.

## Introduction

The cytomegaloviruses (CMV) are a family of species-specific viruses that, after acute infection, persist for the life of the host in a latent form and periodically reactivate. In order to avoid elimination by the host immune system the CMV utilize an array of immune evasion strategies to interfere with the anti-viral response [1]. The capacity of CMV to inhibit apoptosis, which functions as an innate defence mechanism against viral infection, is critical for efficient replication (reviewed in [2]–[3]). The fact that CMV encode multiple cell death inhibitors indicates that the evolutionary survival of this family of viruses relies on the ability to prevent the death of infected cells.

The Bcl-2 family of proteins are important regulators of cell death whose main function is to regulate the integrity of the mitochondrial membrane [4]. The activation of Bax and Bak, two pro-apoptotic Bcl-2 family members, are essential for apoptosis to proceed in many cells types [5]–[6]. In response to apoptotic stimuli Bax and Bak undergo a series of conformational changes and oligomerise before mediating the permeabilisation of the mitochondrial membrane [7]. Permeabilisation of the mitochondrial outer membrane results in pro-apoptotic proteins such as cytochrome *c*, being released into the cytoplasm where they participate in the activation of caspase-dependent and -independent signalling pathways.

Since many forms of apoptosis require the activity of Bax or Bak, it is not surprising that many viruses have evolved strategies to inhibit these proteins. Some viruses such as adenovirus and some γ-herpesviruses prevent apoptosis by encoding homologues of pro-survival Bcl-2 proteins [8]. Despite lacking sequence homologues of Bcl-2, several CMV-encoded proteins capable of inhibiting Bax have been identified. Human CMV (HCMV) encodes a viral mitochondria-localized inhibitor of apoptosis (vMIA) that prevents mitochondrial permeabilization by inhibiting Bax [9]–[13]. More recently, the m38.5 protein, encoded by murine CMV (MCMV), was identified as a functional orthologue of vMIA [14]–[17]. Surprisingly, replication of an m38.5 deletion virus was similar to that of the parental virus in most tissues analysed during *in vivo* infection [17]. Expression of m38.5 was however important in maintaining the viability of infected leukocytes, indicating that during *in vivo* infection MCMV initiates Bax-mediated death in only a sub-set of permissive cells [17].

Under most circumstances the activation of either Bax or Bak is sufficient to induce apoptosis [5], [18]. Since m38.5 is a Bax-specific inhibitor, effective inhibition of cell death by MCMV was predicted to require an inhibitor of Bak. A recent *in vitro* study identified m41.1 as a Bak-specific inhibitor of apoptosis [19]. Macrophages infected with a Δm41.1 mutant virus were more sensitive to apoptosis and m41.1 was found to prevent Bak oligomerisation [19]. The m41.1 protein is encoded entirely within the m41 ORF, but in a different reading frame to m41. The m41 protein product is a Golgi localised protein that has also been implicated as having anti-apoptotic activity [19]–[20]. These finding indicate that multiple anti-apoptotic proteins are derived from the m41 ORF of MCMV.

To date the role of proteins derived from the m41 ORF has been assessed solely by limited *in vitro* studies and the physiological relevance of the proteins encoded within the m41 ORF is unclear. Our analysis revealed that three protein products are produced from the m41 ORF. In addition to m41.1, alternative splicing results in the production of two forms of the m41 protein. By constructing specific deletion mutants we have defined the relative contribution of the m41 proteins to the pathogenesis of MCMV during *in vivo* infection. Replication of an MCMV mutant lacking both forms of m41 was attenuated in the lungs, but equivalent to that of WT virus in other visceral organs. By contrast, growth of a Δm41.1 virus was attenuated in multiple organs in WT mice, but not in Bak-deficient mice. Optimal *in vivo* replication of MCMV therefore relies on the capacity of m41.1 to inhibit Bak-mediated apoptosis.

## Results

### Splicing of m41 transcripts results in the production of two protein products

**Figure 1:**
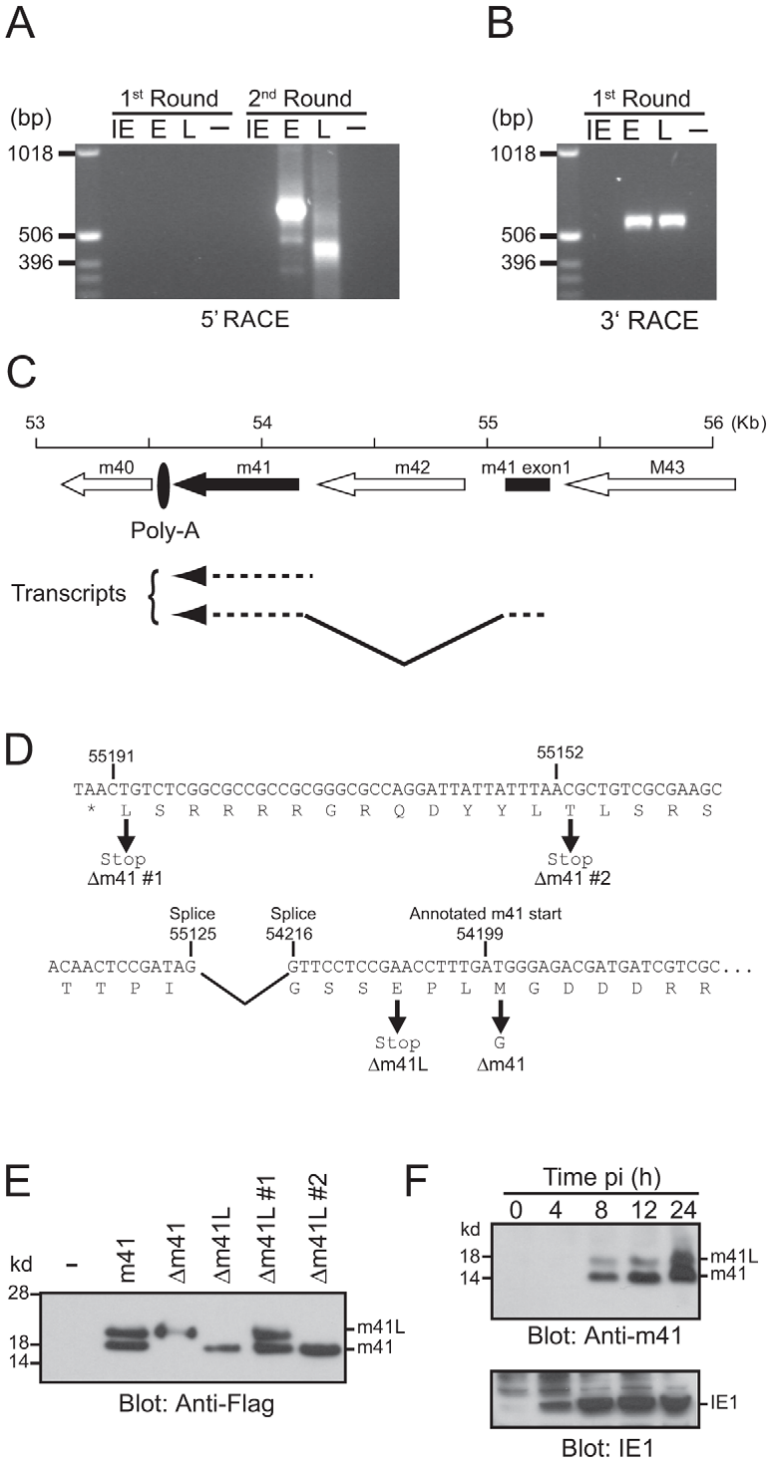
Genomic arrangement and analysis of the m41 locus. RNA isolated from MCMV infected fibroblasts at IE, E and L times post-infection was subjected to (**A**) 5′ RACE or (**B**) 3′ RACE analysis. The resulting products were separated on 1% agarose gels. (**C**) The genomic region of MCMV encompassing the annotated m41 ORF (solid arrow), the newly identified 41 exon (solid box), and adjoining ORFs (open arrows) is shown. Direction of transcription is indicated by the orientation of the arrows. Location of the common polyadenylation site used by all genes in this region is denoted by the filled oval. (**D**) Sequence of the annotated m41 ORF is shown. The annotated m41 translation start site and the location of splice acceptor and donor sites are shown above the DNA sequence. Location of mutations within the m41 ORF are shown below the DNA sequence. (**E**) The indicated m41 constructs were transiently overexpressed in Cos-7 cells, total cell lysates prepared and expression of the m41 and m41L proteins detected by immunoblot using anti-flag antibodies. (**F**) Total cell lysates were prepared from fibroblasts infected with MCMV at the indicated time pi and the expression of m41, m41L, and IE1 proteins detected by immunoblot.

The m41 ORF encodes two protein products, m41 and m41.1, that are proposed to function by inhibiting apoptosis [19]–[20]. However, the relative contribution of these proteins to the pathogenesis of MCMV during *in vivo* infection has not been assessed. Moreover, previous work suggested that two distinct m41 protein products are produced from the m41 ORF, although their origin has not been defined [20]. We therefore utilized 5′ and 3′ rapid amplification of cDNA ends (RACE) to define transcripts produced from the m41 ORF (**Fig. 1 A and B**). Fibroblasts were infected with MCMV and RNA isolated at immediate early (IE), early (E) and late (L) times post-infection (pi). After two rounds of 5′ RACE two major transcripts were identified at E and L times pi (**Fig. 1A**). Sequencing of these transcripts determined that the shorter transcript encompasses the annotated m41 ORF sequence (**Fig. 1C**). The longer transcript is composed of two exons; the first exon is a short sequence originating upstream of the m42 ORF with a splice donor site at position 55,125. The first exon is spliced to the m41 sequence at position 54,216 which is in frame with the annotated m41 start codon (**Fig. 1C and D**). Sequencing of 3′ RACE products indicates that both transcripts terminate at the predicted m41 poly-A site (**Fig. 1 B and C**). Thus, two potential m41 protein products are produced during MCMV infection, the first from the annotated m41 ORF and the second is an N-terminally extended version of m41.

The N-terminally extended m41 transcript does not encode a conventional AUG translation initiation start site (**Fig. 1D**), however, translation initiation can also utilize codons such as CUG and ACG [21]. The larger m41 transcript encodes a number of potential alternative initiation codons that could be used to generate an N-terminally extended m41 protein. In order to determine if an N-terminally extended m41 protein could be produced from the larger RNA transcript, a cDNA construct encoding the transcript was generated and a Flag tag added to the C-terminus. Cos-7 cells were transfected with the m41 construct and total cell lysate immunoblotted with anti-Flag antibodies. Two m41 protein products, which we have denoted m41 and m41L, were detected by immunoblot (**Fig. 1E**). The m41 product is presumably produced by translation commencing at the annotated AUG (54,199) while translation upstream of the annotated m41 AUG yields m41L. We constructed a number of additional m41 cDNA constructs in order to define the translational start sites of the two proteins. A construct lacking m41 (Δm41) was produced by mutating the annotated m41 start methionine to glycine. Transfection of the Δm41 cDNA resulted in the production of the m41L, but not of m41 protein (**Fig. 1 E**). Thus, translation of the m41 protein occurs at the annotated m41 start site of 54,199. A construct lacking m41L (Δm41L) was produced by mutating the codon for glutamic acid located upstream of the m41 start site to a stop codon. Transfection of the Δm41L construct resulted in the production of the m41 protein only (**Fig. 1 E**), confirming that m41L is an N-terminally extended form of m41. Mutation of the potential translation initiation codon at 55,191 (Δm41 #1) did not prevent the production of m41L, however, no m41L protein was detected after transfection of the Δm41 #2 cDNA (**Fig. 1 E**). Both ACG and CUG have been described as non-conventional translation initiation codons, hence, translation of m41L commences at either 55,152 or 55,149 (**Fig. 1D**).

To determine if multiple forms of m41 are produced during viral infection, an anti-m41 polyclonal antibody was produced. Total cell lysates were prepared from fibroblasts infected with MCMV at the indicated times pi and immunoblotted with the anti-41 antibody. Both m41L and m41 proteins were detected as early as 8 h pi in MCMV-infected fibroblasts (**Fig. 1F**). Thus, two distinct m41 protein products are produced during MCMV infection, with m41L being an N-terminally extended form of m41.

### Overexpression of m41.1, but not m41 or m41L suppresses Bak-dependent apoptosis

**Figure 2:**
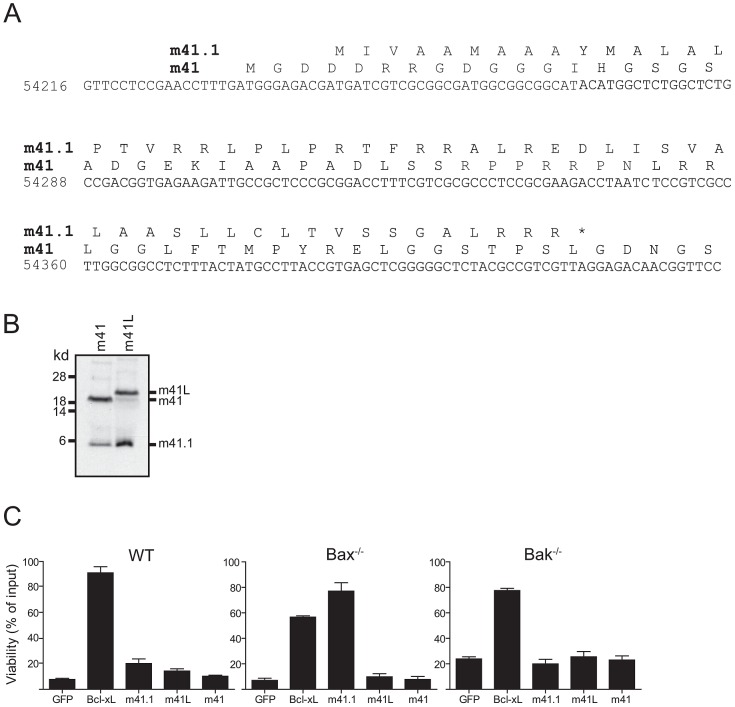
m41.1 encodes a Bak-specific inhibitor. (**A**) The predicted amino acid sequences of m41 and m41.1 in the K181-Perth MCMV strain are shown. (**B**) *In vitro* transcription/translation reactions using cDNA constructs encoding m41 or m41L were performed, the resulting protein products were separated by SDS-PAGE and detected by autoradiography. (**C**) Fibroblasts derived from WT, Bax- or Bak-deficient mice were infected with retroviruses encoding the indicated proteins. Cells were treated with 10 µM staurosporine for 24 hr and cell viability assessed. (n = 6).

The m41.1 protein was recently identified as a Bak-specific inhibitor of apoptosis in the Smith strain of MCMV, and a similar protein was identified in rat CMV [19]. An m41.1 protein is also predicted to be encoded within the 41 ORF of the K181-Perth strain of MCMV used in this study (**Fig. 2A**). The predicted sequence of the K181 derived m41.1 protein is identical to that of the Smith derived protein, with the exception of the seventh amino acid (Ala in K181 versus Thr in Smith) suggesting that the Bak-inhibitory function mediated by m41.1 is retained in the K181-Perth strain of MCMV.

Since an m41.1 specific RNA transcript was not detected in our RACE analysis, we hypothesised that the m41.1 protein is produced from at least one of the m41 RNA transcripts. To test this possibility cDNA constructs encoding m41 or m41L were used in an *in vitro* transcription/translation assay. Proteins of the expected size for m41 and m41L were readily detected in this assay (**Fig. 2B**). Importantly, a protein of the expected size for m41.1 was produced from both m41 constructs (**Fig. 2B**). This finding indicates that translation of m41.1 can occur from either of the m41 RNA transcripts.

The role of m41 during MCMV infection is unclear. Overexpression of a protein equivalent to m41 is not sufficient to inhibit apoptosis [19]. However, an increase in apoptosis was noted when macrophages were infected with a Δm41 deletion virus [19]. Importantly the capacity of m41L to inhibit apoptosis has not been assessed. We therefore tested the ability of m41L to inhibit apoptosis when overexpressed in fibroblasts. WT fibroblasts, or fibroblasts genetically deficient in either Bak or Bax were infected with the indicated retroviral constructs, treated with staurosporine, and cell viability assessed 24 h later by MTT assay. As expected, Bcl-x_L_ inhibited apoptosis in all cell lines tested (**Fig. 2C**). Overexpression of m41.1 inhibited apoptosis in *Bax^−/−^* cells, but not in WT or Bak-deficient cells (**Fig. 2C**). This finding is in agreement with earlier data [19] indicating that m41.1 can inhibit apoptosis mediated by Bak. Conversely, neither m41L nor m41 was capable of inhibiting apoptosis in any of the cell lines tested (**Fig. 2C**). Thus, unlike m41.1, expression of m41L or m41, in isolation, does not prevent cell death mediated by either Bax or Bak.

### Proteins derived from the m41 ORF enhance viral replication *in vivo*

**Figure 3:**
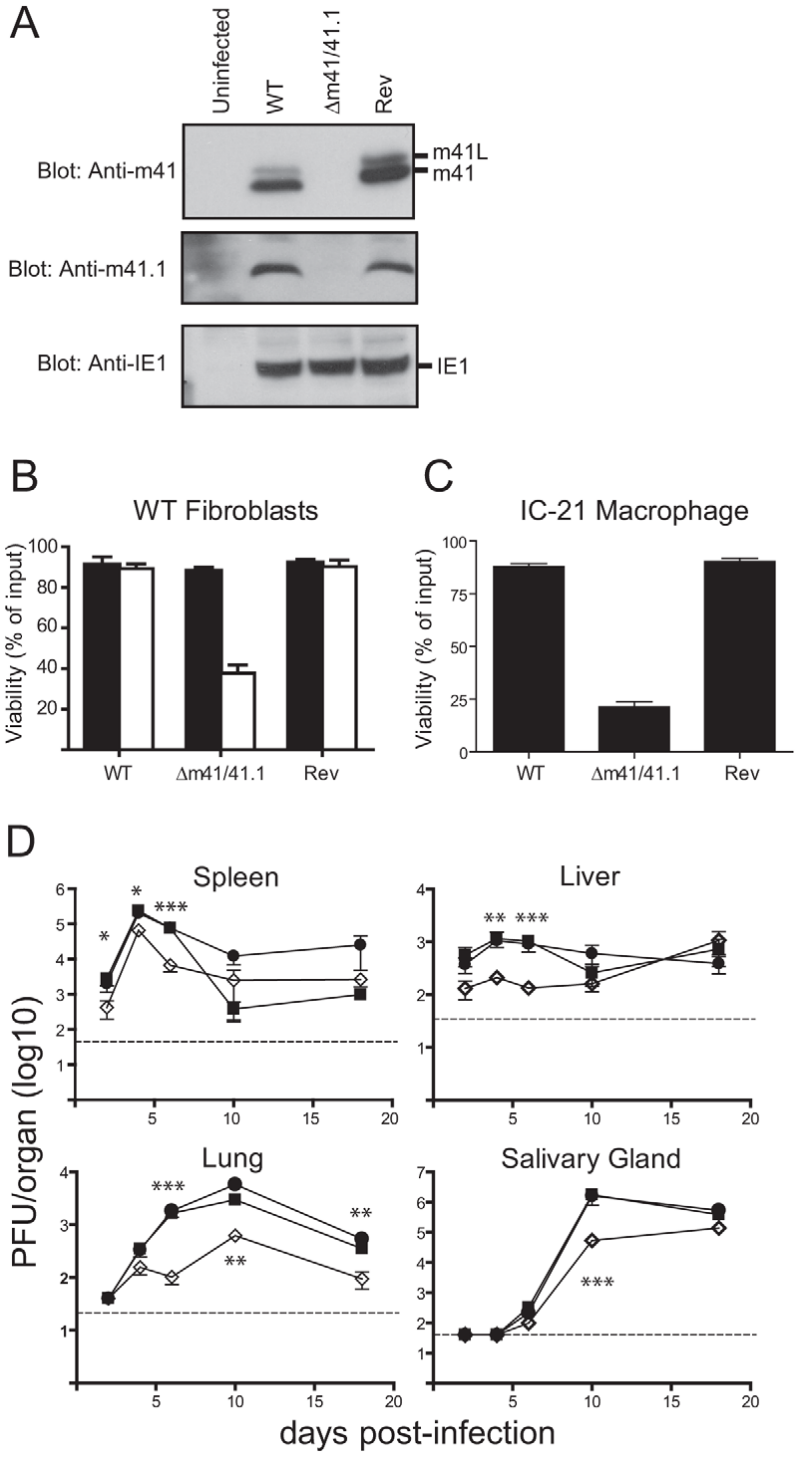
Mutation of the m41 ORF inhibits viral replication. (**A**) Fibroblasts were infected with WT MCMV, the Δm41/m41.1 mutant or a revertant (Rev) virus at an MOI = 3. Total cell lysates were prepared at 24 hr pi and immunoblotted with anti-m41, anti-m41.1, or anti-IE1 antibodies. (**B**) Fibroblasts were infected with the indicated viruses (MOI = 3) and 18 hr later treated with 100 µM etoposide (open columns) or vehicle (filled columns). Cell viability was quantified by Trypan Blue exclusion 24 hr later (n = 4) (**C**) IC-21 macrophages were infected with the indicated viruses (MOI = 3) and cell viability determined 48 hr pi (n = 4) (**D**) BALB/c mice were infected with WT MCMV (filled square), Rev virus (filled circle), or Δm41/m41.1 (open diamond), organs were removed at the indicated times pi and viral load per organ determined by plaque assay. Viral titers were quantified in three separate experiments and the data pooled, mean ± S.D. of 6–9 mice per group is plotted. * *P*<0.05, ** *P*<0.005, *** *P*<0.0001. Dotted line indicates the limit of detection of the assay.

To evaluate the role of the m41 proteins during infection we constructed a mutant virus termed Δm41/41.1 that does not produce any of the m41 proteins. The Δm41/41.1 virus was generated by introducing a stop codon in-frame with the m41 coding sequence such that Asp4 of m41 was mutated to a stop. The mutation of the m41 sequence also results in the mutation of the m41.1 sequence such that Met1 is mutated to Leu. These changes were predicted to prevent the expression of the m41 proteins and m41.1. Western blot analysis of fibroblasts infected with the Δm41/41.1 virus confirmed that m41, m41L and m41.1 proteins were not expressed (**Fig. 3A**). A revertant (Rev) virus was produced by repairing the mutation within the m41 ORF. Comparing the revertant virus to the Δm41/41.1 mutant ensures that any growth defects detected in subsequent analysis are due to loss of the m41 proteins and not the result of mutations elsewhere in the viral genome. We have previously demonstrated that MCMV-infected cells are resistant to apoptosis initiated by stimuli such as growth factor withdrawal or cytotoxic drugs [17], [22]. As expected the viability of fibroblasts infected with either WT or Rev virus was not affected by etoposide treatment (**Fig. 3B**). By contrast, addition of etoposide to fibroblasts infected with Δm41/41.1 virus resulted in significant cell death (**Fig. 3B**). MCMV is capable of replicating in broad range of cell types, with cells such as macrophages and DCs more sensitive to the absence of anti-apoptotic proteins [17], [23]. We therefore, assessed the impact of infection of the Δm41/41.1 mutant on the viability of IC21 macrophages. Infection of macrophages with WT or Rev virus had no impact on cell viability, while a significant proportion of cells infected with the Δm41/41.1 virus had died by 48 h pi (**Fig. 3C**). Next, the capacity of the Δm41/41.1 virus to replicate *in vivo* was assessed by quantifying viral titers in BALB/c mice. Mice were infected with WT, Δm41/41.1 mutant, or Rev virus and viral titers in the target organs of spleen, liver, lungs and salivary glands determined. Replication of the Δm41/41.1 viral mutant was attenuated in all the organs assessed, with a pronounced defect in viral replication evident in the liver and lungs (**Fig. 3D**). Thus, proteins derived from the m41 ORF promote efficient *in vivo* replication of MCMV.

### m41.1 prevents the premature death of virally infected cells *in vitro*

**Figure 4:**
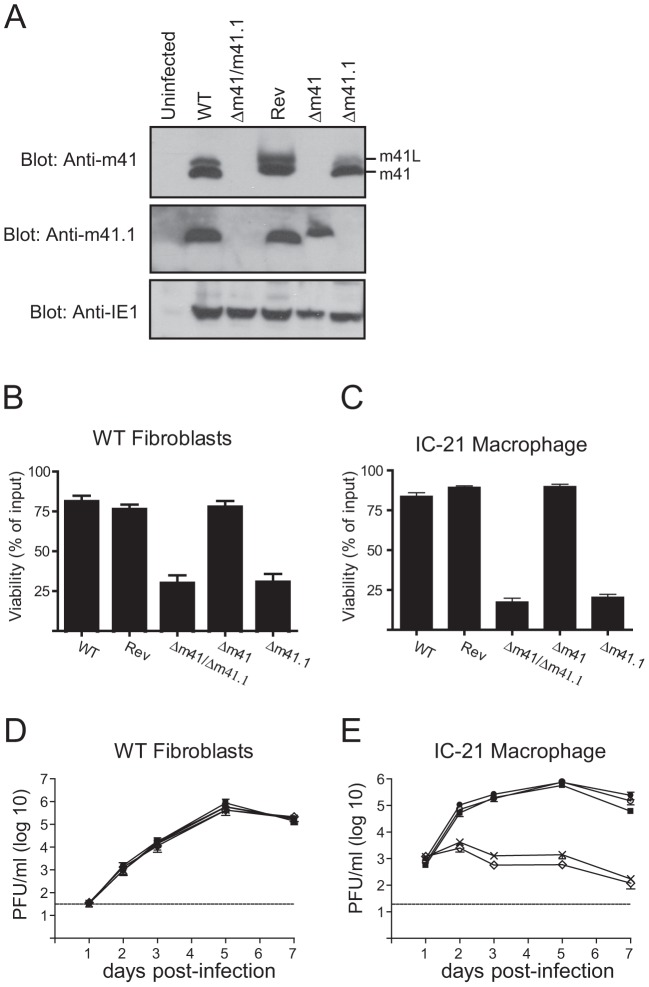
*In vitro* analysis of m41 mutant viruses. (**A**) Fibroblasts were infected with WT, Rev or the indicated MCMV mutants and total cell lysates prepared 24 hr later. Immunoblot analysis was performed using antibodies specific for m41, m41.1 or IE1 as indicated. (**B**) Fibroblasts were infected with the indicated viruses (MOI = 3) and 18 h later 100 µM etoposide added. Cell viability was quantified by Trypan Blue exclusion 24 hr after the addition of etoposide (n = 6). (**C**) IC-21 macrophages were infected with the indicated viruses (MOI = 3) and cell viability assessed 48 hr later (n = 8). (**D**) Fibroblasts or (**E**) IC-21 macrophages were infected with WT MCMV (filled square), Rev (filled circle), Δm41 (open circle), Δm41.1 (cross) or Δm41/m41.1 (open diamond) (MOI = 0.05 for fibroblasts and MOI = 0.5 for IC-21) and viral replication measured at the indicated times pi (n = 6 for fibroblasts and macrophages). Dotted line indicates the limit of detection of the assay.

Having established that the m41 proteins enhance MCMV replication *in vivo*, additional mutants were constructed in order to assess the relative contribution of the m41 proteins, and m41.1, to viral pathogenesis. Mutants specifically lacking expression of m41.1 (termed Δm41.1) or both m41 and m41L (termed Δm41) were constructed using BAC mutagenesis. The Δm41 virus was produced by mutating the annotated m41 ATG to a stop codon, introducing a mutation at this position was predicted to have no impact on the expression of m41.1. Immunoblotting of lysates infected with the Δm41 virus confirmed that expression m41 and m41L was absent while expression of m41.1 was not affected (**Fig. 4A**). The Δm41.1 mutant was constructed by mutating the sequence coding for Leu21 of m41.1 to a stop codon. This mutation results in a silent mutation within the m41 sequence. Again immunoblotting of lysates from infected cells confirmed the specific deletion of m41.1 (**Fig. 4A**). The ability of the mutant viruses to inhibit apoptosis was then tested in fibroblasts. Cells infected with the Δm41 mutant were as resistant to etoposide-induced killing as those infected with WT or Rev MCMV (**Fig. 4B**). Conversely, etoposide treatment of cells infected with the m41.1 deletion virus resulted in cell death equivalent to that observed with the Δm41/41.1 mutant. Similarly, infection of macrophages with the Δm41.1 specific mutant virus resulted in cell death, while the Δm41 mutant had no impact on cell viability (**Fig. 4C**). Deletion of m41.1 or m41 and m41L did not affect viral replication in fibroblasts (**Fig. 4D**). However, in macrophages, deletion of m41.1 resulted in a significant attenuation of viral replication (**Fig. 4E**). These results confirm that m41.1 enhances viral replication *in vitro* by preventing the death of infected cells. Importantly, our data establishes that the ability of MCMV-infected cells to resist apoptosis *in vitro* is not dependent on m41 or m41L.

### The m41.1 and m41 proteins have distinct roles during *in vivo* infection

**Figure 5:**
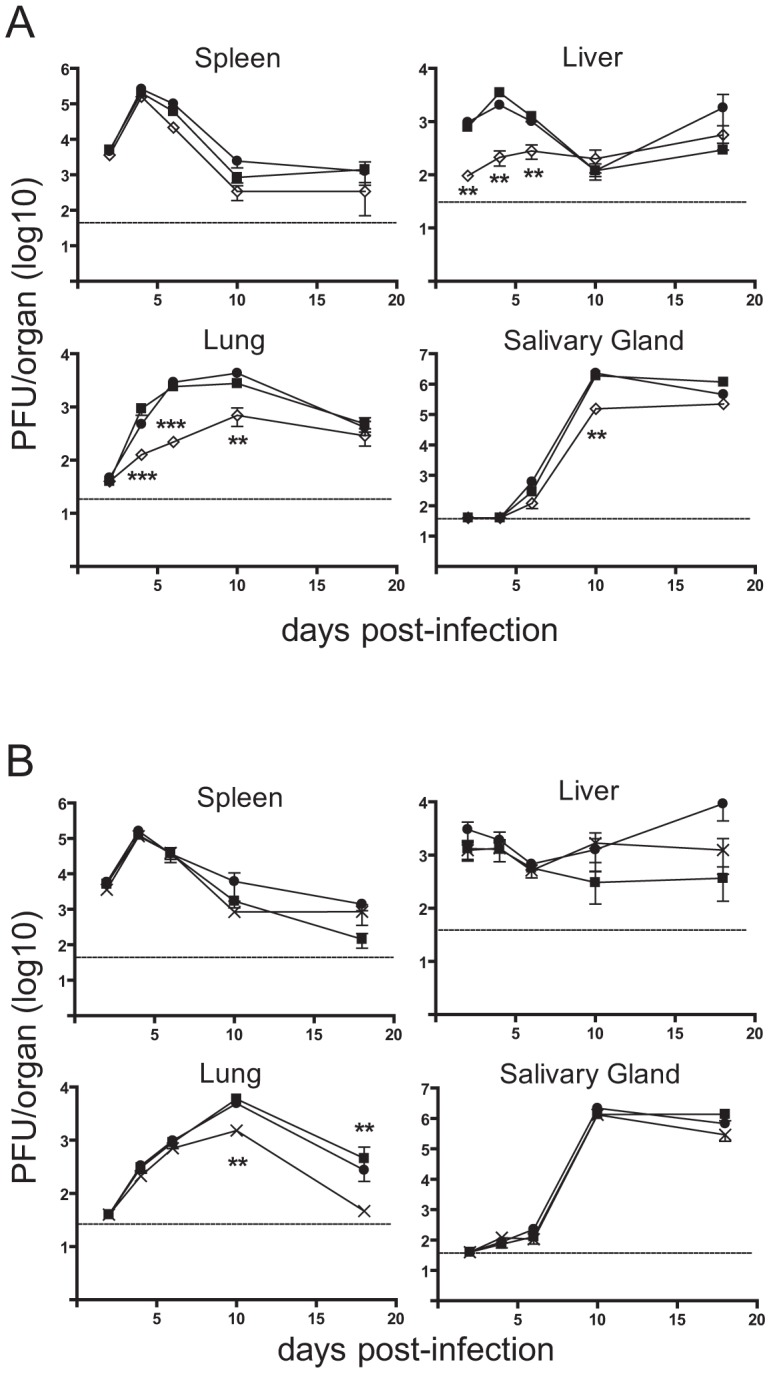
Loss of m41.1 or m41 impairs viral replication *in vivo*. (**A**) BALB/c mice were infected with WT MCMV (filled square), Rev (filled circle) or Δm41.1 mutant (open diamond), organs were removed at the indicated times pi and viral load determined by plaque assay, mean ± S.D. of 5–6 mice per time point is plotted. ** *P*<0.01, *** *P*<0.0001. Dotted line indicates the limit of detection of the assay. (**B**) BALB/c mice were infected with WT MCMV (filled square), Rev (filled circle) or the Δm41 mutant (cross), organs removed at the indicated times pi and viral load determined by plaque assay. Mean ± S.D. of 5–6 mice per time point is plotted. ** *P*<0.01. Dotted line indicates the limit of detection of the assay.

The *in vitro* data suggested that m41.1 inhibits apoptosis, and that this is required for viral replication to proceed in some cell types. The specific contribution of m41.1 to the *in vivo* pathogenesis of MCMV was then assessed by infection of BALB/c mice. Replication of the Δm41.1 mutant in the spleen was indistinguishable from that of WT or Rev virus, and in the salivary gland replication was equivalent to that of the control viruses at all time points with the exception of day 10 pi (**Fig. 5A**). By comparison, growth of the Δm41.1 virus was significantly attenuated in both the liver and lungs at multiple time points. Thus, m41.1 is required for MCMV to replicate effectively in several target organs during *in vivo* infection.

Overexpression of m41L or m41 was not sufficient to inhibit apoptosis (**Fig. 2C**) and a mutant virus lacking both proteins inhibited apoptosis of infected cells as efficiently as the WT virus (**Fig. 4**). Combined, these results suggest that the m41 proteins do not have anti-apoptotic activity. However, given the range of cell types targeted by MCMV during an *in vivo* infection and the diverse means by which apoptosis can be induced we could not exclude the possibility that the m41 proteins inhibit apoptosis during *in vivo* infection. BALB/c mice were therefore infected with the Δm41 mutant and viral replication assessed. Viral titers of the Δm41 mutant were equivalent to those of the control viruses in the spleen, liver and salivary glands at all time points tested (**Fig. 5B**). In the lungs, replication of the Δm41 virus was equivalent to that of the WT virus until day 10 pi when a significant reduction in titre of the mutant virus was noted (**Fig. 5B**). Thus, the m41 proteins are required for optimal replication of MCMV to occur in the lungs.

### m41.1 promotes viral replication *in vivo* by inhibiting Bak

**Figure 6:**
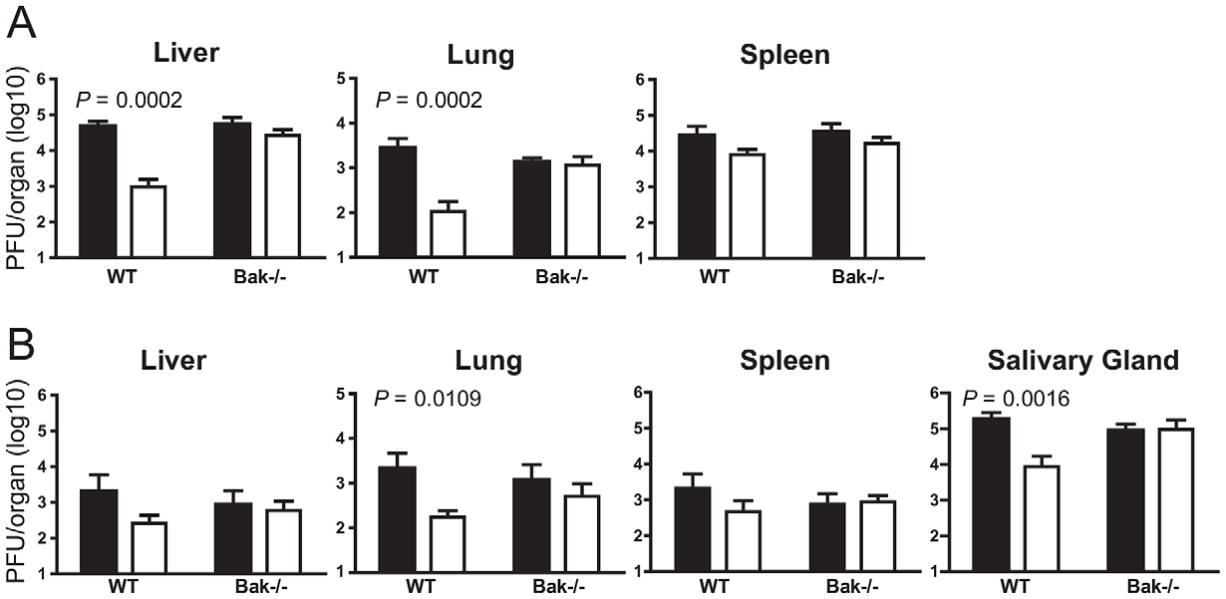
m41.1 inhibits Bak *in vivo*. WT B6 or B6.Bak^−/−^ mice were depleted of NK cells prior to infection with WT MCMV (filled columns) or the Δm41.1 mutant (open columns). Viral load in the indicated organs was quantified by plaque assay on (**A**) day 4 pi, or (**B**) day 8 pi. Mean ± S.D. of 6–8 mice per group is plotted.

The *in vitro* data indicate that m41.1 functions by inhibiting the pro-apoptotic Bak protein. To determine if the role of m41.1 is to prevent Bak-dependent death during *in vivo* infection, mice genetically deficient for Bak were infected with the Δm41.1 mutant. The ability of m41.1 to inhibit Bak-dependent apoptosis was examined in C57BL/6 (B6) mice as Bak-deficient mice on the BALB/c background are not available. B6 mice, unlike the BALB/c strain, are genetically resistant to MCMV due to an effective NK cell response [24]. Therefore Bak-deficient mice, or WT B6 mice, were depleted of NK cells prior to infection with either the WT virus or Δm41.1 mutant. As was the case for BALB/c mice, titers of the Δm41.1 virus were significantly lower than those of the WT virus in the liver and lung at day 4 pi (**Fig. 6A**). By contrast, the titre of the Δm41.1 mutant was equivalent to that of WT MCMV in the spleen at day 4 pi, while, as expected, neither virus was detectable in the salivary gland at this time (**Fig. 6A** **and data not shown**). Furthermore, titers of the Δm41.1 mutant were significantly lower than WT virus in the lung and salivary gland at day 8 pi (**Fig. 6B**). Importantly, replication of the Δm41.1 virus was equivalent to that of the WT virus in B6.Bak^−/−^ mice in all organs tested at both day 4 (**Fig. 6A**) and 8 pi (**Fig. 6B**). These data establish that m41.1 promotes viral replication *in vivo* by inhibiting Bak-dependent killing.

### m41.1 activity promotes viral replication in leukocytes

**Figure 7:**
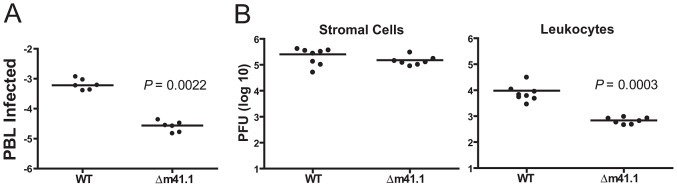
m41.1 enhances viral replication in leukocytes. (**A**) Whole blood was isolated from infected mice on day 5 pi., and red blood cells removed by isotonic lysis. The proportion of infected cells was determined by infectious centre assay. (**B**) Mice were infected with WT MCMV or the Δm41.1 mutant and spleens removed on day 4 pi. Viral burden in stromal cells (left panel), or leukocytes (right panel) were determined by plaque assay.

*In vitro*, replication of the Δm41.1 mutant was significantly attenuated in macrophages while no defect was observed in fibroblasts (**Fig. 4D and E**). To determine if a similar effect occurs *in vivo* we measured virus levels in the blood of infected mice since myeloid progenitors are the predominant cell type infected by MCMV in the blood [25]–[27]. Blood was collected from mice infected with WT MCMV or the Δm41.1 mutant at day 5 pi and the number of infected cells in the circulation determined by infectious centre assay. Mice infected with the Δm41.1 mutant had significantly fewer leukocytes in the blood containing infectious virus (**Fig. 7A**). Thus, m41.1 protects leukocytes in the blood from apoptosis during MCMV infection. We extended this finding by determining if m41.1 enhanced the survival of virally-infected leukocytes in the spleen. In the spleen, MCMV replication occurs in several distinct cell types including cells of the myeloid lineage such as macrophages and dendritic cells (DC), and in splenic stromal cells such as endothelial cells [28]–[29]. Spleens were removed from mice infected with WT or the Δm41.1 mutant at day 4 pi and the viral load within the leukocytes or stromal cell fractions quantified. Viral replication within the leukocyte fraction was significantly reduced in mice infected with the Δm41.1 virus (**Fig. 7B** **, right panel**). However, viral titers of the Δm41.1 mutant were not significantly different from those of the WT virus when viral load within the stromal cell fraction was measured (**Fig. 7B** **, left panel**). Thus, m41.1-mediated inhibition of Bak is required for efficient MCMV replication in leukocytes, but is dispensable for MCMV replication in most permissive cells in the spleen.

## Discussion

Successful replication and transmission of CMV requires the capacity to inhibit or modulate the host immune response and a significant number of viral products are devoted to this purpose. Using a series of mutant viruses we have determined that multiple proteins derived from the m41 ORF enhance MCMV replication *in vivo* by interfering with host anti-viral responses. During *in vivo* infection m41.1 inhibits Bak-mediated apoptosis thereby promoting optimal viral replication. By contrast, m41 and m41L enhance viral replication specifically in the lung. Therefore, the combined actions of proteins derived from the m41 ORF enhance MCMV replication in multiple target organs during *in vivo* infection.

Our *in vitro* data demonstrating that m41.1 functions as a Bak-specific inhibitor of apoptosis is consistent with the findings reported by Cam *et al* [19]. We have expanded on those findings by determining that during *in vivo* infection m41.1 inhibits Bak-dependent apoptosis. We found that m41.1 was required for optimal viral replication in leukocytes isolated from the blood and spleen. A similar defect was noted when the Bax-specific inhibitor m38.5 was deleted from MCMV [17]. Therefore, efficient replication of MCMV in leukocytes requires the simultaneous inhibition of Bax and Bak. By contrast, in other organs inhibition of Bak alone appears sufficient for optimal MCMV replication to proceed. This is particularly evident when replication of the respective MCMV mutants in the liver and lungs is compared. MCMV replication in the liver occurs predominantly in hepatocytes, while in the lungs interstitial fibroblasts, alveolar epithelial cells and endothelial cells are the major sites of viral infection [30]–[31]. Replication of the Δm41.1 mutant was significantly attenuated in the liver and lungs at multiple time points post-infection, whilst we have previously determined that growth of a Δm38.5 virus was equivalent to that of WT MCMV in these organs [17]. Since MCMV replication in hepatocytes and lung parenchymal cells is impaired in the absence of m41.1, but not m38.5, apoptosis induced by MCMV infection in liver and lungs is mediated by Bak, but not Bax. Importantly, the data establish that during viral infection Bax and Bak fulfil cell-type specific functions which are not redundant *in vivo*.

A homologue of m41.1 has been identified in rat CMV, but an obvious sequence homologue does not exist in HCMV [19]. The HCMV vMIA protein, like m38.5, is a Bax-specific inhibitor of apoptosis suggesting that an m41.1-like protein may be present in HCMV. While m38.5 and vMIA perform a similar function, the sequence identity between them is weak. This fact prompted us to test several HCMV ORFs that are located in a similar position in the HCMV genome to m41.1 for their capacity to inhibit apoptosis despite their lack of homology to m41.1. Overexpression of UL41a from the AD169 strain could not inhibit Bak-dependent apoptosis in murine fibroblasts and no protein product was detected when a UL41 construct was transfected (data not shown). These findings do not preclude the possibility that HCMV encodes an m41.1 homologue, but suggest that if a homologue exists, its location within the viral genome differs from that of MCMV. Alternatively, HCMV may not require a Bak-specific inhibitor due to the action of vMIA. A number of publications have demonstrated that vMIA functions as a Bax-specific inhibitor [12]–[13], [16], however, the ability of vMIA to inhibit Bak has been noted [32]. Expression of vMIA may therefore be sufficient to inhibit both Bax and Bak thus eliminating the requirement for HCMV to encode an m41.1 homologue.

In addition to encoding an inhibitor of apoptosis, we have shown that the m41 ORF encodes two additional proteins that have immune evasion properties. In our hands neither form of m41 exhibited any anti-apoptotic activity when overexpressed in fibroblasts. Similarly, cells infected with the Δm41 mutant *in vitro* were as resistant to apoptosis as those infected with the WT virus. However, others have reported that infection of macrophages with a m41 deletion mutant results in an increased rate of apoptosis [19]. The different methods used to construct the mutant viruses may account for the divergent results. The Δm41 virus used in the present study was produced by mutating the m41 ATG codon, thus expression of m41.1 expression is under the control of the endogenous promoter. By contrast, the m41 deletion virus used by Cam et al was produced by deleting the entire m41 ORF and then inserting the m41.1 sequence under the control of the phosphoglycerate kinase promoter in an ectopic position in the viral genome [19]. The increased in apoptosis observed when cells were infected with this mutant could therefore be the result of altered expression of m41.1, rather than a direct effect mediated by the m41 proteins. Alternatively, the differences in strains of MCMV used to construct the respective m41 mutants could account for the different findings.

While lack of m41 expression had no measurable impact on MCMV replication *in vitro*, during *in vivo* infection, replication of the Δm41 mutant was attenuated at late time points post-infection in the lungs. The finding that growth of the Δm41 mutant is attenuated only at late times after infection suggests that improved control of this mutant virus by the host immune system is occurring. Given that m41 is localized to the Golgi apparatus [20], the m41 proteins may interfere with the function of antigen presenting cells (APC). CMV infection is known to modulate the adaptive anti-viral immune response by impairing the surface expression of a variety of molecules, such as major histocompatability complex (MHC) class I and II, CD80, and CD86 [29], [33]–[35]. Our preliminary data indicate that the m41 proteins do not target any of the obvious candidate molecules since macrophages infected with the Δm41 virus down-regulate MHC class I and II, CD86, CD80 and CD54 as efficiently as cells infected with WT virus (data not shown). Monitoring the generation of T cell responses in mice infected with the Δm41 virus will help in establishing if the m41 proteins impede APC functionality. Alternatively, the m41 proteins may directly inhibit T cell mediated killing of virally infected cells, for example, by inhibiting perforin mediated killing of infected cells. How the m41 proteins mediate their effects is currently being investigated.

In addition to improving our understanding of the factors regulating MCMV replication, analysing the *in vivo* growth of MCMV has provided important insights into the regulation of basic cellular processes. Our current study has provided compelling evidence that m41.1 promotes MCMV replication by inhibiting Bak. Furthermore, we have established that m41 and/or m41L proteins are required for optimal viral replication in the lungs. Thus, the m41ORF encodes several distinct immune-evasion proteins that are required for optimal *in vivo* replication of MCMV.

## Materials and Methods

### Ethics statement

All animal experimentation was performed with the approval of the Animal Ethics and Experimentation Committee of the University of Western Australia and according to the guidelines of the National Health and Medical Research Council of Australia.

### Cell lines and reagents

Culture conditions for fibroblast and macrophages, and methods for the purification and titration of viral stocks have been described previously [22], [29]. COS-7 cells were transfected with pcDNA3 encoding Flag tagged m41 constructs using Fugene-6 reagent (Roche, Switzerland) and total cell lysates prepared 48 h later using CHAPS detergent according to published methods [22]. Expression of the various m41 proteins during viral infection was assessed by infecting fibroblasts with WT or m41 deletion mutants at an MOI = 3. Infected fibroblasts were lysed at the indicated times pi, total cellular protein isolated and immunoblot analysis performed as described [22]. The m41 rabbit polyclonal antibody was produced by AbSolutions (Perth, Western Australia, Australia) using m41S protein as the immunogen. The m41.1 rabbit polyclonal antibody was produced by GenScript (Piscataway, NJ) using a peptide with the sequence PLPRTFRRALREDL linked to KLH. Retroviral-mediated transfection of fibroblasts was performed as described [36]. Apoptosis was induced by the addition of 10 µM staurosporine (Sigma-Aldrich, St. Louis, MO or 100 µM etoposide (Sigma-Aldrich, St. Louis, MO) as indicated in the figure legend. Quantification of cell viability by trypan blue exclusion was performed as described [22]. Cell viability using the MTT assay was performed using a Cell Growth Determination Kit (Sigma-Aldrich, St. Louis, MO) according to the manufacturer's instructions. The transcription/translation assays were performed using the TnT T7 coupled reticulocyte lysates system (Promega, Madison, WI).

### 3′ and 5′ rapid amplification of cDNA ends

Murine embryonic fibroblasts (MEF) were infected at a multiplicity of infection (MOI) of 5. Infections were performed either in the presence of cyclohexamide (Sigma-Aldrich, St. Louis, MO) (50 µg/ml) for immediate early RNA or phosphonoacetic acid (Sigma-Aldrich, St. Louis, MO) (20 µg/ml) for early RNA, and RNA isolation performed 4 h pi. For the collection of late RNA transcripts, MEF were infected in the absence of metabolic inhibitors and harvested 24 h pi. All RNA extractions were performed using Trizol™ (Life Technologies, Grand Island, NY) according to the manufacterer's instructions. 3′ and 5′ RACE PCR was performed using total RNA prepared at immediate early, early and late times post infection using the FirstChoice RLM-RACE kit (Life Technologies, Grand Island, NY) and m41 specific oligonucleotides. Reactions were carried out according to manufacturer's instructions. The cDNAs obtained were cloned into pGEMT-Easy and sequenced.

### Generation of m41 and m41.1 constructs

Flag tagged m41 constructs and various mutants thereof were amplified by PCR or SEW-PCR and cloned into pcDNA3Flag expression vector (Life Technologies, Grand Island, NY). DNA sequencing was employed to ensure the fidelity of the PCR product. The primer sequences and DNA templates used to generate each of the constructs are listed in **Table S1 and S4**. Retroviral constructs that allowed for the specific expression of m41.1, m41L or m41S in isolation were generated by PCR or SEW-PCR and the resulting products cloned into the pMIG retroviral vector. The DNA templates and PCR primers used to generate each construct is outlined in **Table S2 and S4**.

### Viral mutagenesis

The construction of viruses that disrupt expression of m41 and m41L and/or m41.1 was performed using the ‘BAC recombineering’ method [37]. Briefly, the p*GalK* plasmid was used as a template with primers 1 and 2 (See **Table S4** for primer sequence) to generate a PCR product containing the *Galk* gene flanked by 50 bp of homology to the m41 target sequence. The resulting m41 *GalK* PCR product was transformed into SW102 *E.coli* containing the pARK25-K181 BAC [38] and plated onto minimal media containing galactose to select for recombinants containing the *GalK* gene inserted into the m41 ORF, the resulting construct was denoted pARK25-m41*GalK*. PCR products containing mutations in the m41 or m41.1 sequence were produced using the SEW-PCR technique (DNA template and primers listed in **Table S3 and S4**). These PCR products were transformed into the SW102 containing pARK25-m41*GalK* and recombinants containing the mutated m41 or m41.1 sequences in place of *GalK* were selected on minimal media containing glycerol and 2-deoxy-galactose. A revertant was made from the SW102 pARK25-m41*GalK*, replacing the *GalK* with a WT K181 m41 PCR product in place of the mutated m41 and m41.1 PCR products. The recombinant BACs were streaked to give single colonies and then the m41 region sequenced to ensure that they contained the expected sequence. The recombinant BAC DNA was prepared using PureLink Plasmid Midiprep Kit (Life Technologies, Grand Island, NY) and RFLP analysis performed to ensure no gross mutations had occurred in the genome (**Fig. S1**). The reconstitution of viral progeny from the recombinant BACs was carried out using the purified BAC DNA and fibroblasts derived from *Bax^−/−^Bak^−/−^* mice as previously described [17].

### Analysis of viral growth

Inbred BALB/c or C57BL/6 mice at 8 weeks of age were obtained from the Animal Resources Centre (Perth, Western Australia). C57BL/6.*Bak^−/−^* mice [5], [39] were bred at the Walter and Eliza Hall Institute for Medical Research (Melbourne, Victoria, Australia). Mice were maintained in specific pathogen-free conditions at the Animal Services Facility of the University of Western Australia. Mice were injected intraperitoneally with 1×10^4^ plaque forming units (PFU) of salivary gland-propagated virus stock of WT BAC-derived MCMV-K181, Rev virus or the indicated m41 deletion viruses diluted in phosphate buffered saline containing 0.5% FBS. At various times after infection mice were killed and organs removed for analysis. Viral titres were quantified by plaque assay on monolayers of permissive cells [40]. NK cells were depleted by the administration of 250 µg of the anti-NK1.1 specific monoclonal antibody PK136 on days −2, 0 and 2 relative to MCMV infection. CD4 T cells were depleted using 500 µg of the anti-CD4 specific monoclonal antibody GK1.5 antibody per delivery and CD8 T cells were depleted using 250 µg of the anti-CD8β monoclonal antibody 53.5.8 per delivery. Mice were injected with T cell depleting antibodies on days −2, 0, 2 and 6 pi. Specific depletion of cell populations was confirmed by FACS analysis. Viremia in the blood was assessed by isolating blood by heart puncture, and determining the number of infected cells by culture on permissive cells [26]. Leukocytes and stromal cells from the spleen were isolated as described [17], [28]. Briefly, spleens were passed through a 70 µm filter, the filter was then extensively washed with sterile medium. Cells retained on the filter, enriched for stromal cells, were lysed by freezing in medium. Cells passing through the filter were retained as the leukocyte fraction. FACS analysis indicated that >90% of the cells passing through the filter were leukocytes. Viral replication in the leukocyte and stromal cell fractions was assessed by plaque assay.

### Statistical analysis

All plotted data represent mean ± standard deviation. All *P* values were determined using the nonparametric Mann-Whitney statistical test.

## Supporting Information

Figure S1 RFLP analysis of MCMV and associated mutants used in this study. Viral DNA from indicated MCMV strains was purified and digested with EcoR1 enzyme. The resulting products were separated by electrophoresis on a 1.5% agarose gel and stained with ethidium bromide.
(EPS)

Table S1 Primers and templates used to generate the various m41 constructs are listed. ^1^ Primer number refers to primers listed in Table S4.
(DOCX)

Table S2 A summary of the primers and DNA templates used to generate the listed m41 retroviral constructs used in this study. ^1^ Primer number refers to primers listed in Table S4.
(DOCX)

Table S3 DNA templates and PCR primers used for the generation of MCMV mutants are listed. ^1^ Primer number refers to primers listed in Table S4.
(DOCX)

Table S4 Complete DNA sequence of all PCR primers used in the study.
(DOCX)
