# Supplementary material for: MCMV-mediated Inhibition of the Pro-apoptotic Bak Protein Is Required for Optimal In Vivo Replication
Source: PLoS Pathog. 2013 Feb 28;9(2):e1003192. doi: 10.1371/journal.ppat.1003192 (PMC3585157; doi:10.1371/journal.ppat.1003192)
Supplement: Table S1 — Primers and templates used to generate the various m41 constructs are listed. 1 Primer number refers to primers listed in Table S4. (DOCX) [file ppat.1003192.s002.docx]

**Table S1. Construction of m41-Flag tagged constructs**

| **Construct** | **1^st^ Round PCR** | | **2nd Round PCR** | |
| --- | --- | --- | --- | --- |
|  | **DNA Template** | **PCR Primer ^1^** | **DNA Template** | **PCR Primer ^1^** |
| pcDNA3-m41 | K181-Perth | 16 and 18 |  |  |
| pcDNA3-Δm41 | K181-Perth | Reaction1: 16 & 28 Reaction 2: 18 & 27 | 1^st^ Round products | 16 & 18 |
| pcDNA3-Δm41L | K181-Perth | Reaction1: 16 & 26 Reaction 2: 18 & 25 | 1^st^ Round products | 16 & 18 |
| pcDNA3-Δm41#1 | K181-Perth | 22 & 18 |  |  |
| pcDNA3-Δm41#2 | K181-Perth | 23 & 18 |  |  |
