# Supplementary material for: MCMV-mediated Inhibition of the Pro-apoptotic Bak Protein Is Required for Optimal In Vivo Replication
Source: PLoS Pathog. 2013 Feb 28;9(2):e1003192. doi: 10.1371/journal.ppat.1003192 (PMC3585157; doi:10.1371/journal.ppat.1003192)
Supplement: Table S2 — A summary of the primers and DNA templates used to generate the listed m41 retroviral constructs used in this study. 1 Primer number refers to primers listed in Table S4. (DOCX) [file ppat.1003192.s003.docx]

**Table S2. Construction of m41 and m41.1 retroviral expression constructs**

| **Construct** | **1^st^ Round PCR** | | **2nd Round PCR** | |
| --- | --- | --- | --- | --- |
|  | **DNA Template** | **PCR Primer ^1^** | **DNA Template** | **PCR Primer ^1^** |
| pMIG-m41L | pcDNA3-Δm41 | Reaction1: 16 & 12 Reaction 2: 19 & 11 | 1^st^ Round products | 16 & 19 |
| pMIG-m41 | K181-Perth | 17 & 19 |  |  |
| pMIG-m41.1 | K181-Perth | 1. & 4 |  |  |
