# Supplementary material for: MCMV-mediated Inhibition of the Pro-apoptotic Bak Protein Is Required for Optimal In Vivo Replication
Source: PLoS Pathog. 2013 Feb 28;9(2):e1003192. doi: 10.1371/journal.ppat.1003192 (PMC3585157; doi:10.1371/journal.ppat.1003192)
Supplement: Table S3 — DNA templates and PCR primers used for the generation of MCMV mutants are listed. 1 Primer number refers to primers listed in Table S4. (DOCX) [file ppat.1003192.s004.docx]

**Table S3. Construction of MCMV mutants**

| **Virus** | **1^st^ Round PCR** | | **2nd Round PCR** | |
| --- | --- | --- | --- | --- |
|  | **DNA Template** | **PCR Primer ^1^** | **DNA Template** | **PCR Primer ^1^** |
| Δm41/m41.1 | K181-Perth | Reaction1: 16 & 6 Reaction 2: 18 & 5 | 1^st^ Round products | 3 & 4 |
| Δm41.1 |  | Reaction1: 16 & 12 Reaction 2: 18 & 11 | 1^st^ Round products | 3 & 4 |
| Δm41 | K181-Perth | Reaction1: 16 & 8 Reaction 2: 18 & 7 | 1^st^ Round products | 3 & 4 |
| Rev | K181-Perth | 3 & 4 |  |  |
