# Supplementary material for: MCMV-mediated Inhibition of the Pro-apoptotic Bak Protein Is Required for Optimal In Vivo Replication
Source: PLoS Pathog. 2013 Feb 28;9(2):e1003192. doi: 10.1371/journal.ppat.1003192 (PMC3585157; doi:10.1371/journal.ppat.1003192)
Supplement: Table S4 — Complete DNA sequence of all PCR primers used in the study. (DOCX) [file ppat.1003192.s005.docx]

**Table S4. PCR primer sequences**

| **Primer** | **Sequence 5’ to 3’** |
| --- | --- |
| 1. m41-Galk-F | CCGTTTCCTCACATTCCGTTGTCGTGCGCAGGTTCCTCCGAACCTTTGcctgttgacaattaatcatcggca |
| 1. m41-GalK-R | ATGACGATGCAGATCACTGCGAGGACGGAGGCGTAAACCACGTCGGGAtcagcactgtcctgctcctt |
| 1. m41-50bp-F | CCGTTTCCTCACATTCCGTTGTCGTGCGCAGGTTCCTCCGAACCTTTG |
| 1. m41 R2 | atgacgatgcagatcactgcg |
| 1. m41-Stop-F | GGAGACTAAGATCGTCGCGGCGAT |
| 1. m41-Stop-R | ACGATCTTAGTCTCCCATCAAAGG |
| 1. m41-M2*-Fwd | CCTTTGTAGGGAGACGATGATCGT |
| 1. m41-M2*-Rev | GTCTCCCTACAAAGGTTCGGAGGA |
| 11. m41.1-L21to*-Fwd | AGAAGATAGCCGCTCCCGCGGACC |
| 12. m41.1-L21to*-Rev | GAGCGGCTATCTTCTCACCGTCGG |
| 16. m41ex1Fwd | Ttcaagcttgctgtgtctgagagtctc |
| 17. m41Fwd | tttaagcttaccatgggagacgatgatcgtc |
| 18. m41Rev | Aaagaattcgtctgtcaatgatcacgac |
| 19. M41RevSTOP | aaatctagatcatctgtcaatgatcacgac |
| 20. M41.1-F-BamH1 | tttggatccGACGATGATCGTCGCGGC |
| 22. m41-ex1-StopA | CCGAAGCTTATAGTCTCGGCGC |
| 23. m41-ex1-StopB | CCGAAGCTTATAGCTGTCGCGAAGCACAA |
| 25. m41-L-Stop-F | TCCTCCTAACCTTTGATGGGAGAC |
| 26. m41-L-Stop-R | CAAAGGTTAGGAGGAACCTATCGG |
| 27. m41-L-only-F | CCTTTGGGGGGAGACGATGATCGT |
| 28. m41-L-only-R | TCTCCCCCCAAAGGTTCGGAGGAA |
